# Effects of Brownfield Remediation on Total Gaseous Mercury Concentrations in an Urban Landscape

**DOI:** 10.3390/s20020387

**Published:** 2020-01-10

**Authors:** Linghui Meng, Charles T. Driscoll, Mario Montesdeoca, Huiting Mao

**Affiliations:** 1 Department of Civil and Environmental Engineering, Syracuse University, Syracuse, NY 13244, USA; ctdrisco@syr.edu (C.T.D.); mmontesd@syr.edu (M.M.); 2 Department of Chemistry, College of Environmental Science and Forestry, State University of New York, Syracuse, NY 13210, USA; hmao@esf.edu

**Keywords:** total gaseous mercury concentrations, diurnal and seasonal variation, brownfield, urban

## Abstract

In order to obtain a better perspective of the impacts of brownfields on the land–atmosphere exchange of mercury in urban areas, total gaseous mercury (TGM) was measured at two heights (1.8 m and 42.7 m) prior to 2011–2012 and after 2015–2016 for the remediation of a brownfield and installation of a parking lot adjacent to the Syracuse Center of Excellence in Syracuse, NY, USA. Prior to brownfield remediation, the annual average TGM concentrations were 1.6 ± 0.6 and 1.4 ± 0.4 $\mathrm{ng}\cdot m^{-3}$ at the ground and upper heights, respectively. After brownfield remediation, the annual average TGM concentrations decreased by 32% and 22% at the ground and the upper height, respectively. Mercury soil flux measurements during summer after remediation showed net TGM deposition of 1.7 $\mathrm{ng}\cdot m^{-2}\cdot {\mathrm{day}}^{-1}$ suggesting that the site transitioned from a mercury source to a net mercury sink. Measurements from the Atmospheric Mercury Network (AMNet) indicate that there was no regional decrease in TGM concentrations during the study period. This study demonstrates that evasion from mercury-contaminated soil significantly increased local TGM concentrations, which was subsequently mitigated after soil restoration. Considering the large number of brownfields, they may be an important source of mercury emissions source to local urban ecosystems and warrant future study at additional locations.

## 1. Introduction

Atmospheric cycling of mercury in urban landscapes contrasts with that in rural environments. Industrial activities, fossil fuel combustion, and human use release large amounts of mercury directly into the atmosphere increasing local mercury concentrations. Mercury soil evasion in urban areas can be lower than rural areas due to an abundance of impervious land cover which limits evasion. Gabriel et al. (2006) [1] showed that mercury evasion rate from pavement surface in Tuscaloosa, Alabama (0.02 ng m^−2^ h^−1^) is much lower than bare soil (6.48 ng m^−2^ h^−1^). The urban heat island effect can result in local secondary circulation, preventing transport of mercury to the upper atmosphere. Urban structures also interrupt air exchange. Weakened air exchange associated with the heterogeneous distribution of mercury sources can result in the remarkable spatial variation of atmospheric mercury concentrations within areas of a city. Carpi and Chen (2002) and Kim et al. (2011) [2,3] observed marked spatial variation of gaseous elemental mercury (GEM) within New York City, USA and Seoul, Korea, respectively.

There have been few studies on the impact of brownfield remediation on ambient concentrations of total gaseous mercury (TGM). A brownfield is a former industrial site where redevelopment or reuse may be complicated by the presence of contaminants. According to the U.S. Environmental Protection Agency, there are more than 450,000 brownfields in the U.S (https://www.epa.gov/brownfields/brownfield-overview-and-definition). Brownfields represent potentially important sources of mercury contamination in urban areas. However, limited research has been conducted on the evasion of mercury from brownfields and their influence on local concentrations of TGM. From a review of the literature, we developed the following research questions: Are brownfields a source of local mercury emissions? If so, how do these affect the temporal patterns of TGM concentrations over adjacent areas? Are brownfield removal and site remediation effective in decreasing local TGM concentrations?

To address these questions, TGM concentrations were measured at two heights (1.8 and 42.3 m) adjacent to a brownfield in Syracuse, NY, USA. Measurements were made over two periods: prior to 2011–2012 and after 2015–2016 soil restoration. Soil restoration involved surface soil removal and installation of a large impervious area (parking lot). Soil mercury evasion measurements were also conducted in June 2015 immediately after brownfield removal to evaluate the soil–sink behavior of TGM. TGM variation at the site was examined over diurnal and seasonal timescales and compared to other urban and rural sites in New York State. 

## 2. Materials and Methods

### 2.1. Site Description

This study was conducted at the Urban Ecological Observation Tower (43°3′0.05″ N, 76°8′25.62″ W) at the Syracuse Center of Excellence (CoE) in Syracuse, NY, USA. The site of CoE was the former location of a typewriter manufacturing plant beginning in 1903, which continued to manufacture at the site until 1962. Subsequently, the plant was converted to a commercial building known as the Midtown Plaza. The building was demolished in 1999 with funding from the Voluntary Cleanup Program administered by the New York State Department of Environmental Conservation (which was replaced by the Brownfield Cleanup Program in 2003). The foundations and basement were left in the ground, and the basement cavity was filled with debris from the building demolition. The surface of the site was finished with crushed stone and used as a surface parking lot. In 2004, New York State and Syracuse University selected the site for construction of the headquarters of the Syracuse Center of Excellence for Environmental and Energy Systems. Construction of CoE building began in 2006. Construction activities included remediation for a variety of contaminates. The CoE headquarters building was opened in 2010. 

**Figure 1:**
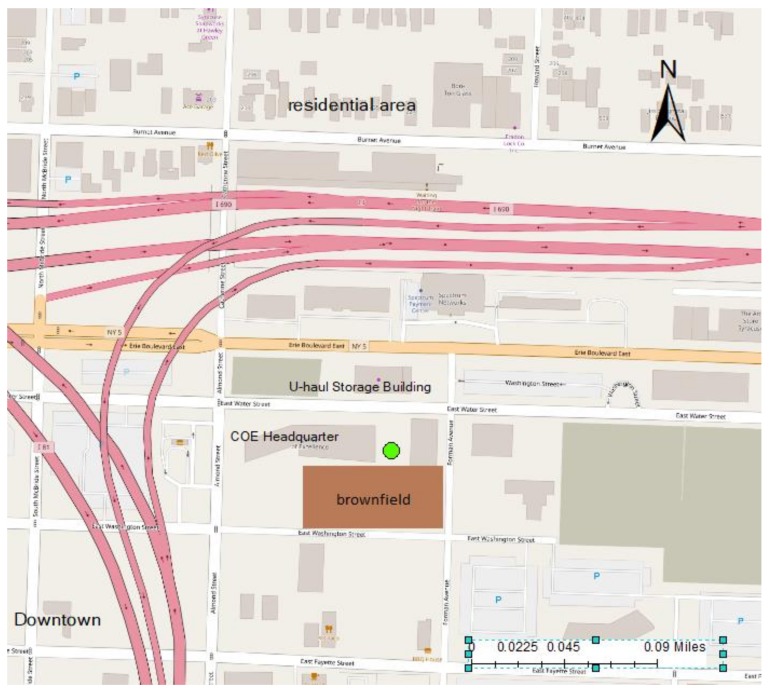
Sampling location of total gaseous mercury measurements at green circle in Syracuse, NY, USA. Interstate highways are shown in pink. The location of the former brownfield site is shown in brown.

The site is located at the urban core of Syracuse, adjacent to two major highways (U.S. routes 81 and 690; Figure 1). Commercial buildings are positioned to the south and residential houses to the north of the tower. The dominant land cover is impervious land, comprising 68% of the total area of 1 km^2^ around the tower [4]. There was a large area of brownfield adjacent to the tower, and approximately 135,000 tons of contaminated soil were removed in May 2015 for site restoration and parking lot construction (4950 m^2^). Mercury concentrations in the soil removed from the site ranged from 0.05 to 0.23 $\mathrm{mg}\cdot g^{-1}$ (number of observation (n) = 18).

Syracuse is the fourth most populous city of New York State (144,700; 2013 Census). The region has a humid continental climate. The annual average temperature is 9.4 °C, annual rainfall is 977 mm, and annual snowfall is 3145 mm (National Climatic Data Center, www.ncdc.noaa.gov).

### 2.2. Sampling Methods

#### 2.2.1. Atmospheric Mercury 

**Figure 2:**
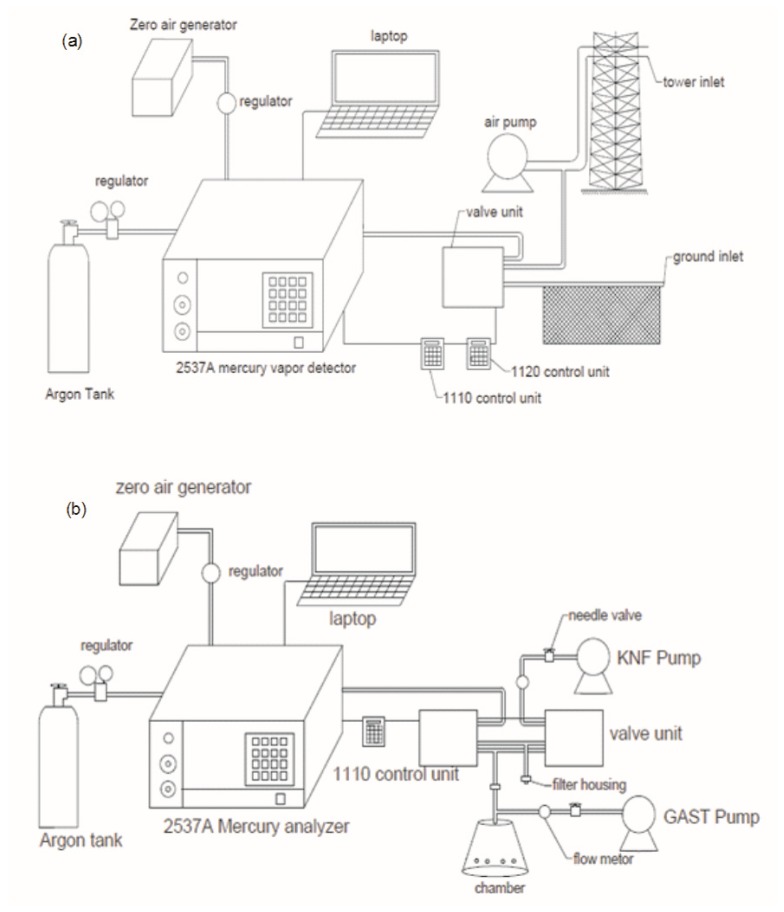
Schematic diagram of atmospheric total gaseous mercury (TGM) measurement system (**a**) and soil evasion measurement system (**b**) used in this study.

TGM concentrations in air were measured at two heights: ground and the upper tower level (Figure 2a). The sampling inlet at the ground level is 1.8 m above the land surface adjacent to the observation tower, next to E. Water Street. The sampling inlet of the upper level is located on the top of the observation tower (42.3 m). A pump was used to deliver air samples from the top of the tower and the ground level to a continuous mercury analyzer. Duplicate measurements were taken at each inlet and then switched via a valve controlled by a two-port controller to sample the other inlet (described below). A controller was also used for standard additions for quality control measurements. TGM concentrations were measured over three periods (June 2011–July 2011, November 2011–June 2012, June 2015—May 2016).

#### 2.2.2. Mercury Flux Measurements 

Soil mercury evasion was measured using a dynamic flux chamber during June 2015 (Figure 2b). The dynamic flux chamber is a 3.87 L clear polycarbonate vessel, 18.4 cm in diameter, which was sealed to the ground surface. To facilitate the air exchange rate in the chamber, 8 holes equally distanced 5 cm above the ground surface are positioned on the chamber wall. A Tekran 2537A mercury vapor analyzer was used to make separate measurements of TGM concentrations inside the dynamic flux chamber and outside ambient air. One inlet connected to the top of the chamber was used to sample air inside the chamber. The other inlet was positioned next to the chamber, 5 cm above the ground surface. Duplicate measurements were taken at each inlet and switched via a valve unit controlled by a two-port controller. Soil mercury evasion flux is calculated as follows (Equation (1)):

(1) $$F=\left(C_{\mathrm{chamber}}-C_{\mathrm{ambient}}\right)\times \frac{Q}{A}$$

F is mercury flux rate ($\mathrm{ng}\cdot m^{-2}\cdot h^{-1}$);
C_chamber_ is the TGM concentration of air inside the flux chamber ($\mathrm{ng}\cdot m^{-3}$);
C_ambient_ is the TGM concentrations of ambient air ($\mathrm{ng}\cdot m^{-3}$);
Q is flow rate of flushing air ($L\cdot \min^{-1}$); and
A is the area of soil exposed in the chamber (m^2^).

In this study, Q was 4.4 $L\cdot \min^{-1}$, and A was 0.0266 m^2^. Two pumps were used in TGM measurements. One pump (3.4 $L\cdot \min^{-1}$) was used to accelerate air extraction from the chamber when measuring TGM concentration inside the chamber. The second pump ($4.4\;L\cdot \min^{-1}$) was used to maintain air movement inside the chamber when the analyzer was measuring TGM concentrations of ambient air. 

### 2.3. Quality Assurance/Quality Control

#### 2.3.1. Automatic Calibration

The Tekran 2537A mercury vapor analyzer can achieve automatic calibration. In this study, automatic calibration was made every 25 h.

#### 2.3.2. Standard Addition

The performance of the Tekran 2537A mercury analyzer was verified with the standard addition of a permanent source inside the analyzer. The recovery rate of the standard addition is used to determine the performance of the analyzer. The analyzer is regarded as working effectively if the recovery rate falls within the range of 80–120%. In this study, the standard addition occurred after every 35 measurements and the recovery rate was calculated with Equation (2).

(2) $$R=\frac{C_{\mathrm{measured}}-C_{\mathrm{background}}}{C_{\mathrm{theory}}}\times 100\%$$

C_measured_ is the actual mercury concentration detected by the 2537A analyzer ($\mathrm{ng}\cdot m^{-3}$);
C_background_ is the mercury concentration in ambient air ($\mathrm{ng}\cdot m^{-3}$);
C_theory_ is the theoretical mercury concentration ($\mathrm{ng}\cdot m^{-3}$).

In this study, C_theory_ was 27.2 $\mathrm{ng}\cdot m^{-3}$.

#### 2.3.3. Manual Injection

The manual injection was used to examine the accuracy of the soil evasion flux system. Saturated mercury vapor was injected into the chamber through holes on the chamber wall. The mercury concentration was measured using the analyzer and the recovery rate of mercury was calculated with Equation (3). In this study, the mercury analysis system is regarded as providing accurate measurement within 80–120% of the recovery rate.

(3) $$R=\frac{\left(C_{\mathrm{measured}}-C_{\mathrm{background}}\right)\times T_{\mathrm{measured}}\times Q}{C_{\mathrm{saturation}}\times V_{\mathrm{injected}}}$$

C_saturation_ is the mercury saturation concentration at a given air temperature ($\mathrm{ng}\cdot m^{-3}$);
C_measured_ is the mercury concentration the 2537A analyzer detected ($\mathrm{ng}\cdot m^{-3}$);
C_background_ is the mercury concentration in ambient air ($\mathrm{ng}\cdot m^{-3}$);
T_measured_ is the time of sampling air measured by the 2537A analyzer (min);
V_injection_ is the volume of saturate mercury air injected in the sampling air flow (μL);
Q is the flow rate of flushing air ($L\cdot \min^{-1}$);

In this study, C_saturation_ is quote from mercury saturation concentration table based on air temperature, T_measured_ was 5 min, Q is 4.4 $L\cdot \min^{-1}$.

### 2.4. Supporting Data

Meteorological data were available from a weather station installed on the tower and operated by the State University of New York College of Environmental Science and Forestry (SUNY-ESF). These data include air temperature (at 1.8 and 42.3 m), relative humidity, solar radiation, precipitation, wind speed, and direction at 15-min intervals. TGM data at the Syracuse CoE were compared to values from other sites, including a site operated by Dr. Huiting Mao located on the roof of Jahn Hall on the SUNY-ESF campus, which is approximately 25 m above ground and located 1.8 km south of the COE site (McGowan et al., 2017) and three monitoring sites from the National Atmospheric Deposition Program Atmospheric Mercury Network (AMNet; http://nadp.slh.wisc.edu/amnet/default.aspx ), located in Rochester, NY (43°8′46.67″ N, 77°32′53.20″ W), Huntington Forest in the rural Adirondacks (43°58′23.16″ N, 74°13′23.16″ W), and in New York City (40°52′4.80″ N, 73°52′41.52″ W).

### 2.5. Data Analysis

The mean value and standard deviation of the measurements for a particular period were calculated using data within the 90th confidence interval of that period. The data were classified by season (winter: December–February; spring: March–May; summer: June–August; and fall: September–November) and hours of the day (Eastern Standard Time). The daily average air temperature, solar radiation, and TGM concentrations were calculated and relationships were evaluated by regression analysis. The wind data were classified into 11 subsets in 1 m/s intervals, and average TGM concentrations for each subset were calculated. Spearman’s correlation coefficient was used in a bivariate correlation analysis. *t*-test and ANOVA test were used to examine differences in variables between two and among more groups, respectively. Linear regression was used to examine linear relationships between factors. 

## 3. Results

### 3.1. Overall and Seasonal Characteristics of TGM

**Figure 3:**
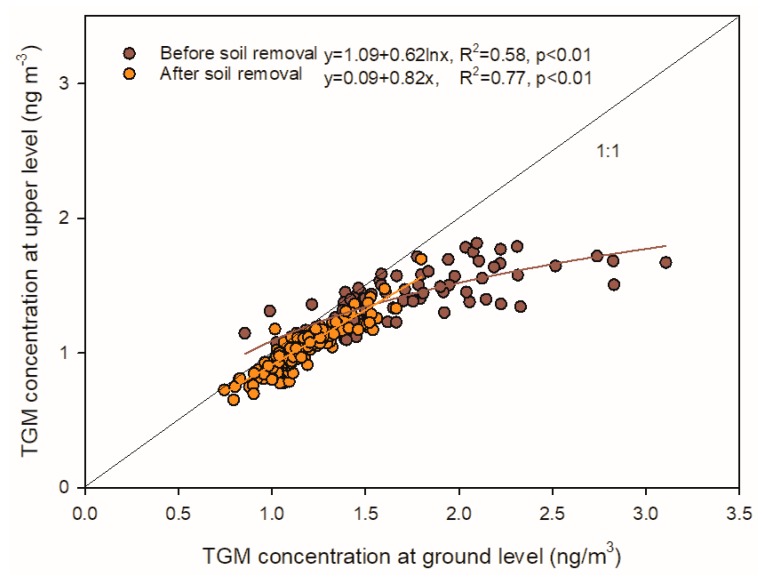
Comparison of daily average concentration at two heights (1.8 and 42.3 m) prior to and after brownfield remediation at the Syracuse CoE.

The average TGM concentrations at the Syracuse CoE site prior to brownfield remediation were 1.6 ± 0.6 and 1.4 ± 0.4 $\mathrm{ng}\cdot m^{-3}$ at the ground and upper (42.3 m) levels, respectively (Table 1). TGM concentrations at the ground level were significantly higher than those at the upper level during this period (*p* < 0.01). TGM concentrations at the upper level generally increased with increasing TGM concentrations at the ground level (Figure 3). At low TGM concentrations (<1.5 $\mathrm{ng}\cdot m^{-3}$), values at the upper level (y) approached but were significantly lower than ground level values. However, as concentrations increased at the ground level (>1.5 $\mathrm{ng}\cdot m^{-3}$), values at the upper level also increased but to a lesser degree than ground level values, resulting in an increasing concentration difference between the two sampling heights (y = 1.09 + 0.62·lnx, *R*^2^ = 0.58, *p* < 0.01). After brownfield remediation and parking lot installation, significant decreases in TGM concentrations were observed at both heights. The annual average TGM concentrations were 1.1 ± 0.3 and 1.1 ± 0.2 $\mathrm{ng}\cdot m^{-3}$ at the ground and upper level, respectively (Table 1). There was no significant difference between TGM concentrations at the two heights following brownfield remediation (y=0.91x+0.18, *R*^2^ = 0.88, *p* < 0.01: where x is TGM concentrations at the ground level and y is TGM concentration at the upper level).

Prior to soil remediation, the seasonal average concentrations of TGM were 1.5 ± 0.4, 1.8 ± 0.6, 1.7 ± 0.6, and 1.4 ± 0.3 $\mathrm{ng}\cdot m^{-3}$ at the ground level and 1.3 ± 0.3, 1.5 ± 0.3, 1.4 ± 0.5, and 1.3 ± 0.3 ${\mathrm{ng}\;m}^{-3}$ at the upper level in spring, summer, fall, and winter, respectively (Table 1). The maximum mean TGM concentrations at both heights occurred in summer, and the minimum occurred in winter. After soil remediation, seasonal average concentrations of TGM were 1.2 ± 0.2, 1.1 ± 0.3, and 1.1 ± 0.3 $\mathrm{ng}\cdot m^{-3}$ at the ground level in spring, summer, and fall, respectively; and 1.1 ± 0.1, 1.0 ± 0.3, 1.0 ± 0.3, and 1.2 ± 0.1 $\mathrm{ng}\cdot m^{-3}$ at the upper level in spring, summer, fall, and winter, respectively (Table 1), with ground level values for spring, summer, and fall significantly higher than upper level values. Note that data were not available for the ground level in winter after remediation. The maximum TGM concentrations at the upper height after brownfield remediation occurred in winter, in contrast to the maximum seasonal value during summer prior to remediation.

### 3.2. Diurnal Variation in Different Seasons

**Figure 4:**
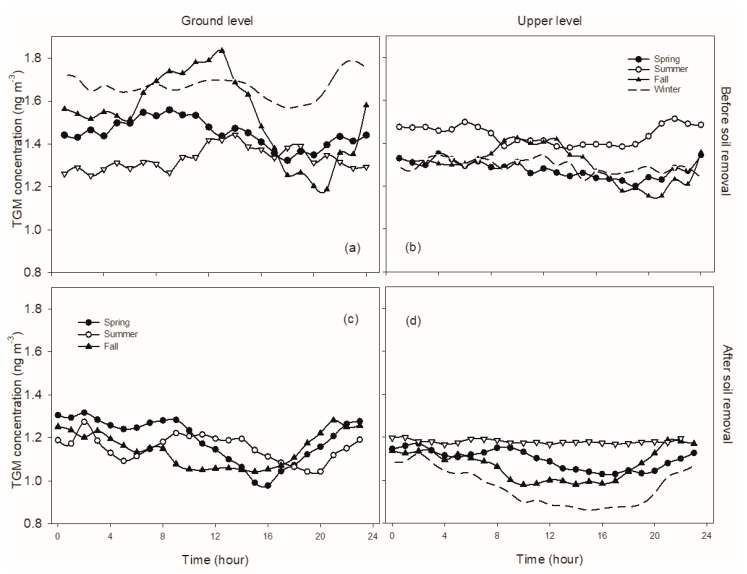
Comparison of diurnal patterns of TGM concentration at ground level (**a**,**c**) and height (**b**,**d**) before (2011–2012; (**a**,**b**)) and after brownfield remediation (2015–2016; (**c**,**d**)) at the Syracuse CoE.

Different diurnal patterns of TGM concentration for different seasons were observed at the ground level prior to brownfield remediation (Figure 4). In spring, TGM occurred at higher concentrations from midnight, increased reaching a maximum concentration at 10:00, decreased until sunset, and then increased in the early evening (Figure 4a). During summer, TGM concentrations increased starting early afternoon reaching a peak at midnight, followed by another increase at sunrise and reaching a maximum in the early morning, and then values decreased until early afternoon (Figure 4b). In fall, TGM concentrations increased after sunrise and reaching a peak at noon, and then decreased in the afternoon, followed by another increase after sunset and reaching a peak before midnight (Figure 4c). During winter, TGM maintained at a low and relatively constant concentration during nighttime, followed by an increase in the day, with values decreasing in the afternoon to the evening (Figure 4d). The daily amplitude was highly variable among seasons, with values greater in spring (0.4 $\mathrm{ng}\cdot m^{-3}$) and fall (0.7 $\mathrm{ng}\cdot m^{-3}$) than summer (0.2 $\mathrm{ng}\cdot m^{-3}$) and winter (0.3 $\mathrm{ng}\cdot m^{-3}$). At the upper height, the diurnal patterns in non-winter seasons were similar to diurnal patterns at the ground level for the same season. But diurnal amplitude was different. The maximum amplitude was observed in summer (0.4 $\mathrm{ng}\cdot m^{-3}$), followed by fall (0.3 $\mathrm{ng}\cdot m^{-3}$), and spring (0.2 $\mathrm{ng}\cdot m^{-3}$). In winter, relatively constant TGM concentrations were observed throughout the day, with a diurnal amplitude of 0.1 $\mathrm{ng}\cdot m^{-3}$ (Figure 4d).

After brownfield remediation, diurnal patterns and amplitude at ground level were similar to those prior to remediation in spring; however, they were significantly altered in fall. During fall, TGM concentrations were higher at nighttime and decreased to lower values during daytime, then increased after sunset. Diurnal amplitude decreased from 0.64 $\mathrm{ng}\cdot m^{-3}$ to 0.24 $\mathrm{ng}\cdot m^{-3}$. During summer, TGM increased after sunset and reaching a maximum at 2:00, followed by a decrease in the early morning, then concentrations increased and remaining elevated until noon, and there was no significant change in diurnal amplitude (from 0.22 to 0.23).At the upper level, TGM had higher concentrations at nighttime and decreased during daytime in non-winter seasons, with diurnal amplitudes of 0.2, 0.4, and 0.4 $\mathrm{ng}\cdot m^{-3}$ in spring, summer, and fall, respectively. TGM concentrations remained at a relatively constant concentration at the upper level throughout the day in winter, with a diurnal amplitude of less than 0.1 $\mathrm{ng}\cdot m^{-3}$. 

### 3.3. Relationships between TGM Concentrations and Meteorological Factors

**Figure 5:**
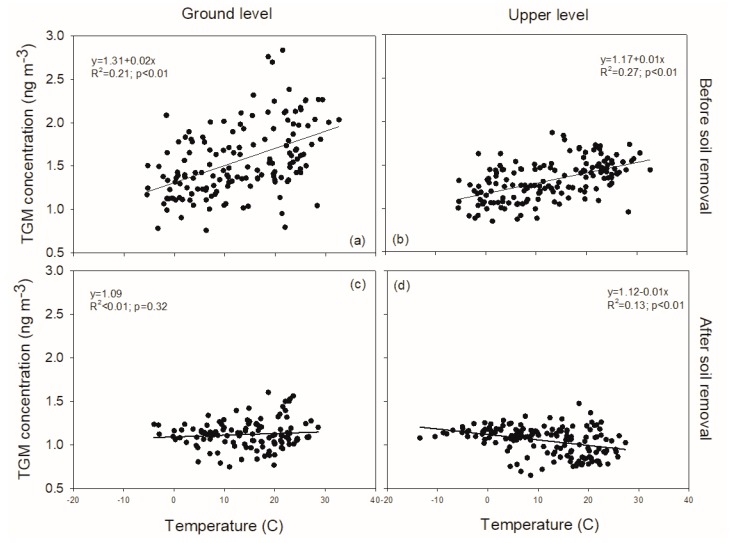
Relationships of TGM concentration with air temperature at ground level (**a**,**c**) and at height (**b**,**d**) before (**a**,**b**) and after (**c**,**d**) brownfield remediation.

The daily average TGM concentrations were positively related with daily average air temperatures at both heights prior to brownfield remediation (Figure 5a,b; $y=1.31+1.97\times 10^{-2}x$; *R*^2^ = 0.21, *p* < 0.01 at the ground level, and $y=1.17+1.19\times 10^{-2}x$; *R*^2^ = 0.27, *p* < 0.01 at upper level, where y is TGM concentration ($\mathrm{ng}\cdot m^{-3}$) and x is air temperature (°C)). After brownfield remediation, no relationship was evident between daily average TGM concentration and air temperature at the ground level (Figure 5c), while a negative linear relationship was observed at the upper height (Figure 5d; TGM concentration (y, in $\mathrm{ng}\cdot m^{-3}$) and air temperature (x, in °C) is $y=1.12-6.31\times 10^{-3}$ x; *R*^2^ = 0.13; *p* < 0.01).

The daily average TGM concentrations were positively correlated with daily average solar radiation at both heights prior to brownfield remediation:

$$y=1.25+1.5\times 10^{-3}x;\;{R^{2}}^{\;}=\;0.12;\;p\;<\;0.01\;(\mathrm{ground}\;\mathrm{level})$$

$$y=1.13+1.0\times 10^{-3}x;\;R^{2}=\;0.18;\;p\;<\;0.01\;(\mathrm{upper}\;\mathrm{level})$$

where y is TGM concentration ($\mathrm{ng}\cdot m^{-3}$) and x is solar radiation (W m^−2^)). No significant correlation between TGM concentrations and solar radiation was observed after brownfield remediation.

**Figure 6:**
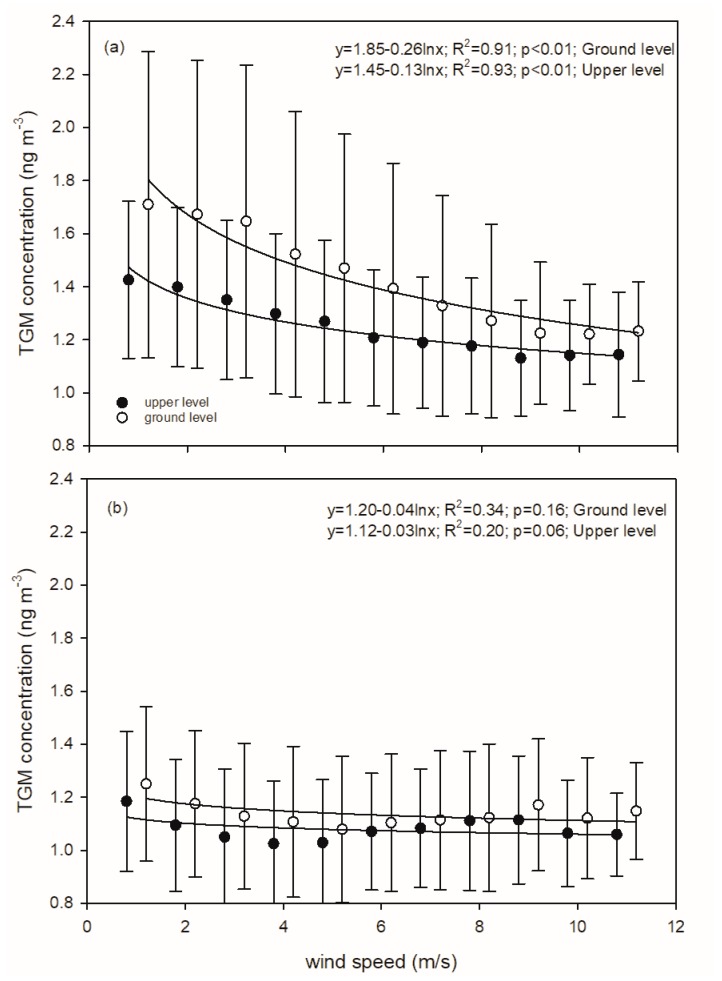
Comparison of the relationship of TGM concentrations with wind speed at the two heights prior to (**a**) and after (**b**) brownfield remediation at the Syracuse CoE.

TGM concentrations were negatively related to wind speed at both heights before soil remediation. TGM concentrations decreased with increasing wind speed at low wind speeds (<7 m/s), approaching a relatively constant concentration with increasing wind speed (Figure 6; y=1.85−0.26·lnx; *R*^2^ = 0.91; *p* < 0.01 at the ground level and y=1.45−0.13·lnx; *R*^2^ = 0.93; *p* < 0.01 at the upper level, where y is TGM concentration ($\mathrm{ng}\cdot m^{-3}$) and x is wind speed (m/s)). After soil remediation and parking lot installation, no relationship between TGM concentrations and wind speed was evident (Figure 6).

### 3.4. Relationship of Mercury Evasion Flux with TGM Concentrations and Meteorological Factors

**Figure 7:**
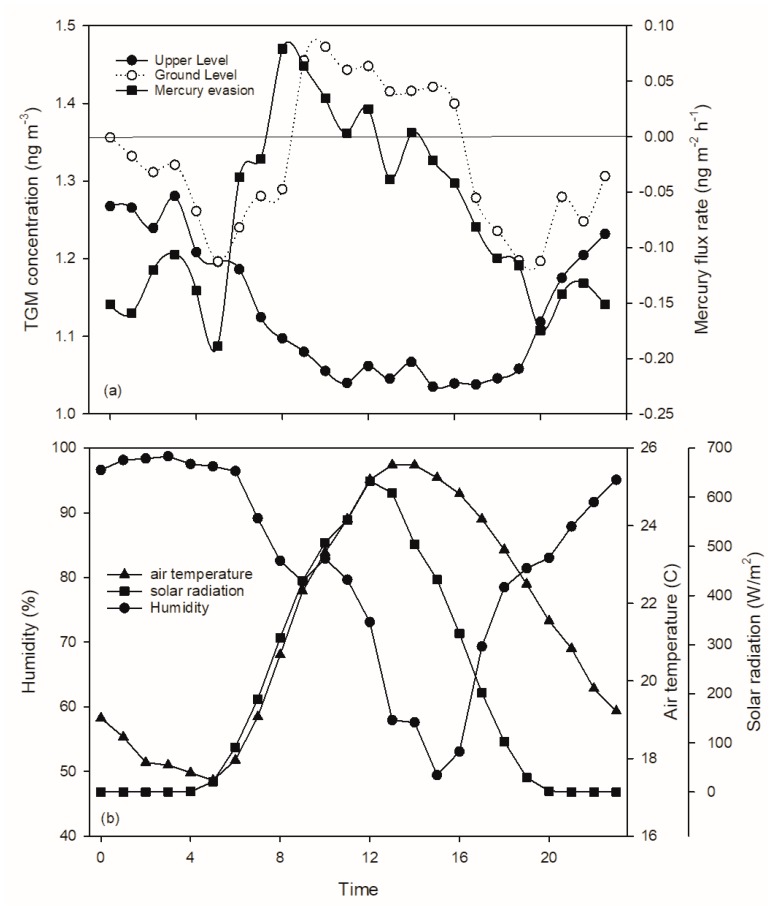
Diurnal pattern of TGM concentrations at the ground level and at height with mercury evasion flux from soil in June 2015 at the Syracuse CoE (**a**). Values are mean over the observations of the month. Shown also are air temperature, solar radiation, and relative humidity (**b**).

Soil mercury evasion flux was measured at the CoE in June 2015 after remediation (Figure 7a). The net daily mercury flux was negative (−1.7 $\mathrm{ng}\cdot m^{-2}\cdot {\mathrm{day}}^{-1}\;;\mathrm{net}\;\mathrm{deposition}$) over the month of measurements. Mercury evasion from soil was negative at night (i.e., net deposition). Values sharply increased at 5:00 reaching a maximum at 8:00, remained as a net evasion to the atmosphere until 12:00, and then decreased to negative values. This flux pattern was positively correlated with the diurnal pattern of TGM concentrations at the ground level for this month (*R* = 0.71, *p* < 0.01), and positively correlated with air temperature (*R* = 0.54, *p* < 0.01) and solar radiation (*R* = 0.83, *p* < 0.01).

## 4. Discussion

### 4.1. Temporal and Spatial Variation in TGM Concentration 

#### 4.1.1. TGM Concentrations Prior to and after Brownfield Remediation

TGM concentrations showed seasonal differences at the Syracuse CoE prior to brownfield remediation, with the highest values in summer and the lowest values in winter (Table 1). This seasonal pattern is thought to be the result of local mercury soil evasion enhanced by warm temperature and strong solar radiation [5,6,7]. This hypothesis is supported by positive correlations between TGM concentrations with air temperature (Figure 6) and solar radiation prior to brownfield remediation. These relationships provide evidence that local mercury evasion drove the magnitude and some of the variation in local TGM concentrations at the CoE before site remediation. 

Significant decreases in TGM concentration were evident at the CoE after brownfield remediation and parking lot installation, with concentrations decreasing 32% and 22% at the ground and upper levels, respectively. There are two plausible explanations for this decrease: a decrease in mercury concentrations due to regional decreases in emissions or decreases associated with decreases in soil mercury evasion after brownfield remediation. The correlations between TGM concentration with air temperature and solar radiation were not evident after remediation, suggesting that local soil evasion was a much smaller contribution to local TGM concentrations after soil restoration. Furthermore, soil mercury flux measurements conducted after brownfield remediation in summer (Figure 7) showed a pattern of net deposition of TGM to this site (−1.7 $\mathrm{ng}\cdot m^{-2}\cdot {\mathrm{day}}^{-1}$), suggesting the site had transitioned from a mercury source to a mercury sink. Note that this rate of net mercury deposition is much lower than values reported for regional forest ecosystems (15.5 μg m^−2^ year^−1^ Huntington Forest, NY [8]; 8.1 μg m^−2^ year^−1^ Hubbard Brook Experimental Forest, NH [9].

#### 4.1.2. Concentration Differences between the Two Heights

Our results revealed significant decreases in TGM concentrations from the ground surface to the upper level prior to brownfield remediation (Figure 3). The vertical TGM concentration gradient is thought to be the reason for this pattern. Because TGM concentrations at the two heights were strongly correlated (*R^2^* = 0.58; *p* < 0.01) and mercury evasion from brownfield was likely an important mercury source prior to remediation, some of the TGM at the upper height was likely derived from local soil emissions and transported vertically to the upper level. Therefore, TGM derived from the ground surface was likely diluted by surrounding air via vertical transport, causing decreases in concentrations at a greater altitude. In addition, faster wind speed at the upper level can facilitate the advection of regional air masses decreasing the influence of the local source (Figure 6). These two factors caused a concentration difference between the two heights. Following brownfield remediation, there were distinct decreases in TGM concentration at both the surface and elevation with no difference in TGM concentrations between the two sampling heights, suggesting a removal of the local mercury source.

#### 4.1.3. Diurnal Variation in TGM Concentrations

**Figure 8:**
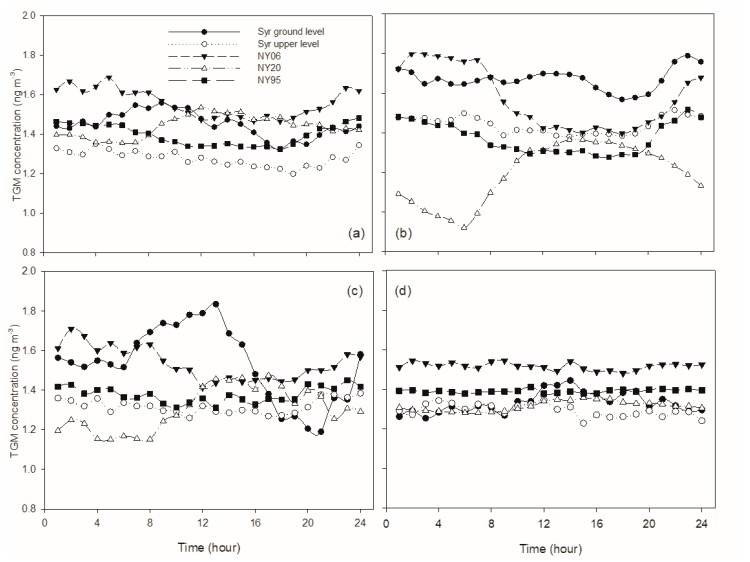
Comparison of seasonal TGM diurnal patterns for Syracuse CoE prior to brownfield remediation with AMNet sites in New York State, including (**a**) spring; (**b**) summer; (**c**) fall; and (**d**) winter (2011–2012).

A diurnal pattern, in which TGM concentrations increase in the morning followed by decreases later during the daytime until the next sunrise, is frequently reported at sites where soil mercury evasion is a significant source [6,10,11], including Huntington Forest (NY20), a forested site (Figure 8). Diurnal variation in TGM at the ground level prior to the soil restoration contrasted with this pattern during the daytime. TGM concentrations were elevated during nighttime in non-winter seasons, but remained at constant lower concentrations during winter. The increase in TGM concentration during nighttime was likely due to the reduced height of the planetary boundary layer (PBL) under constant soil mercury evasion [6,10,12]. The PBL height varies with air temperature, decreasing with decreases in air temperature after sunset, limiting vertical mixing of the surface with upper air, which could result in TGM accumulation near the ground surface. The PBL height increases with increasing air temperature after sunrise, enhancing vertical mixing of TGM. Growth in the PBL height during the day is also associated with increases in the oxidation of gaseous elemental mercury (GEM) [10], which also serves to decrease TGM concentrations. This pattern is commonly observed at urban sites, for example AMNet sites NY06 (Bronx) and NY95 (Rochester) (Figure 8). Local mercury evasion from the brownfield was enhanced with increasing air temperature. The net effect of these factors controls TGM concentrations during daylight.

Winter patterns were different from the non-winter seasons. A nocturnal increase in TGM concentration was not evident at the two heights, and the daytime increase was only observed at ground level prior to soil restoration. Denis et al. (2006) investigated TGM patterns in downtown Toronto and Choi et al. (2013) [10] in Rochester, NY showing that TGM remained constant at low concentrations during nighttime in winter. Denis et al. (2006) [13] observed increases in GEM concentration during daylight at two heights (3.5 and 7 m). Both studies suggested snow cover and air temperature as the reasons for the winter TGM pattern. Moreover, soil mercury evasion is significantly related to air temperature (Gabriel et al., 2006) [1]. Low air temperature in winter likely limited soil mercury evasion at the CoE. Moreover, the ground surface at this site was covered with snow for long periods during winter. These two factors likely restrict soil TGM emissions during winter, and as a result, TGM concentration remained low during nighttime. Increasing mercury evasion with increasing air temperature after sunrise may occur by TGM release from melting snow causing an increase in TGM concentrations. A positive correlation between TGM concentration at the ground level and air temperature during winter (*R*^2^ = 0.12, *p* < 0.01) supports this hypothesis. 

After brownfield remediation, soil mercury evasion at the CoE site decreased and PBL variation likely became a dominant factor influencing diurnal variation. A diurnal pattern, with higher TGM concentrations during nighttime and lower during daytime, was observed during the non-winter seasons at the upper level and in spring and fall at ground level. Even though local soil mercury evasion apparently greatly decreased after brownfield remediation and net mercury deposition was evident from soil flux measurements (Figure 7), there is a period of net evasion of soil mercury in the morning consistent with summer increases in TGM during this period, likely due to diurnal increases in air temperature and solar radiation.

### 4.2. Comparison of TGM Variation at the CoE with Other Sites in NY State

The overall average TGM concentrations from 2011 to 2012 were 1.6 ± 0.4, 1.3 ± 0.3, and 1.4 ± 0.3 $\mathrm{ng}\cdot m^{-3}$ for Bronx (NY06), Huntington Forest (NY20) and Rochester (NY95), respectively, in New York State (Table 1). Ground level TGM at the Syracuse CoE had the highest concentrations among these sites prior to soil restoration, followed by the Bronx, Rochester, the upper level at Syracuse and Huntington Forest. The fact that TGM concentrations at the CoE were higher than those in Bronx and Rochester suggest a strong local source. The overall average TGM concentration from 2015 to 2016 were 1.8 ± 0.3, 1.4 ± 0.2, and 1.2 ± 0.2 $\mathrm{ng}\cdot m^{-3}$ for Bronx, Rochester, and Huntington Forest, respectively. The results show TGM concentrations at Bronx increased by 12.5%, while concentrations at Huntington Forest decreased by 8%, and concentrations at Rochester remained constant from the 2011–2012 value. 

GEM has been reportedly decreasing at a rate of 1.6$\%\cdot {\mathrm{year}}^{-1}$ in Upstate NY (Huntington Forest) consistent with decreases in regional emissions [14]. TGM measurements conducted from 2013 to 2015 at nearby SUNY-ESF [15] showed a 9% decrease in TGM concentrations from the period prior to and after soil restoration. This decrease is likely representative of a regional decrease in Syracuse NY, but the rate is much less than the decrease observed at the CoE site (32% and 22% at the ground and upper level, respectively). Regional decreases in TGM concentrations associated with decreases in regional emissions appear to be too small to explain the abrupt concentration decrease observed at the CoE. The decrease of TGM concentration at the CoE was undoubtedly partially due to decreases of regional background concentrations of TGM, but the remediation was clearly responsible for most of the site-level decrease. 

Different diurnal patterns were observed seasonally at the New York AMNet sites (Figure 8). At Bronx and Rochester, TGM concentrations were higher at nighttime and decreased to lower values during daytime in non-winter seasons. These patterns are consistent with patterns reported by Song et al. (2009) [12] and Lan et al. (2014) [6]. GEM oxidation and variation in the PBL height are considered to be the factors controlling TGM diurnal variation during the non-winter seasons at Bronx and Rochester. Values remained at relatively constant concentrations throughout the day in winter. These patterns are consistent with the winter patterns we observed at the CoE, suggesting a general winter pattern in the urban area of New York State. Reduced solar radiation associated with cold weather conditions and snowpack likely caused TGM to remain at low and constant concentrations during winter at these sites.

At the forested site at Huntington Forest, TGM concentrations increased in the morning, reaching a maximum value around noon, then decreased until next sunrise during non-winter seasons. In winter, TGM concentrations remained constant at night, followed by an increase in concentration in the afternoon. The non-winter patterns at Huntington Forest are similar to patterns reported for other rural areas and are thought to be driven by soil mercury evasion [10,11,16]. In winter, snowpack and cold weather limit mercury evasion and increases in TGM concentrations in the morning were not evident. TGM release by evasion from either soil due to increasing air temperature, or associated with melting snow, may result in increases in TGM concentration in the afternoon. Note that nocturnal TGM increases are characteristic of urban sites in the non-winter seasons, constant anthropogenic mercury emissions are thought to be the mechanism driving nocturnal TGM increases. The lack of nocturnal TGM increases during winter is likely the result of lower air temperature [10,13] or decreases in oxidant concentrations [10]. 

### 4.3. Contributions to Technological Innovation

This study included a number of contributions to technological innovation involving mercury sensor technology. There have been very few measurements of atmospheric mercury in urban areas, particularly focused on brownfields prior to and after remediation. This study shows that soil remediation can be effective at decreasing atmospheric mercury concentrations from local brownfield sources. This study also included detailed temporal measurements of TGM at two vertical locations providing perspective on the impact of this local emission source. Finally, the tower measurements were supported by soil flux measurements after brownfield restoration suggesting that the site may have shifted from a mercury source to a net mercury sink.

## 5. Conclusions

This study provides an assessment of mercury emissions before and after brownfield remediation in urban Syracuse, NY. Our measurements showed that evasion from the brownfield contributed to elevated local TGM concentrations. When contaminated soil was removed and the brownfield was remediated, there was a distinct decrease in local TGM concentrations. Diurnal patterns and relationships of TGM concentrations with meteorological factors are consistent with the contribution from brownfield emissions. These results suggest that brownfields can influence the atmospheric mercury cycle in urban landscapes. Considering the number of brownfields in urban areas and the impact the CoE brownfield had on local TGM concentrations, the effects of brownfields on the mercury cycle of urban ecosystems should be investigated further. 

## Figures and Tables

**Table 1:** Annual mean and standard deviation of TGM concentration for Syracuse Center of Excellence (CoE) and AMNet sites in New York State (in $\mathrm{ng}\cdot m^{-3}$). NY06 is located in Bronx, NY; NY20 is located at Huntington Forest, NY; NY95 is located in Rochester, NY.

		NY06	NY20	NY95	Syracuse Ground	Syracuse Upper
2011–2012	Overall	1.6 ± 0.4	1.3 ± 0.3	1.4 ± 0.3	1.6 ± 0.6	1.4 ± 0.4
	Spring	1.5 ± 0.3	1.5 ± 0.3	1.3 ± 0.2	1.5 ± 0.4	1.3 ± 0.3
	Summer	1.6 ± 0.4	1.2 ± 0.3	1.3 ± 0.2	1.8 ± 0.6	1.5 ± 0.3
	Fall	1.6 ± 0.6	1.2 ± 0.3	1.4 ± 0.5	1.7 ± 0.6	1.4 ± 0.5
	Winter	1.5 ± 0.3	1.3 ± 0.1	1.4 ± 0.2	1.4 ± 0.3	1.3 ± 0.3
2015–2016	Overall	1.8 ± 0.3	1.2 ± 0.2	1.4 ± 0.2	1.1 ± 0.3	1.1 ± 0.2
	Spring	1.8 ± 0.3	1.3 ± 0.1	1.4 ± 0.2	1.2 ± 0.2	1.1 ± 0.1
	Summer	1.8 ± 0.3	1.1 ± 0.2	1.4 ± 0.3	1.1 ± 0.3	1.0 ± 0.3
	Fall	1.7 ± 0.3	1.1 ± 0.2	1.3 ± 0.2	1.1 ± 0.3	1.0 ± 0.3
	Winter	1.8 ± 0.3	1.3 ± 0.1	1.3 ± 0.2		1.2 ± 0.1
